# The Role of Self-Control and Motivation on Exhaustion in Youth Athletes: A Longitudinal Perspective

**DOI:** 10.3389/fpsyg.2018.02449

**Published:** 2018-12-04

**Authors:** Gro Jordalen, Pierre-Nicolas Lemyre, Bård Erlend Solstad, Andreas Ivarsson

**Affiliations:** ^1^Department of Coaching and Psychology, Norwegian School of Sport Sciences, Oslo, Norway; ^2^Department of Coaching and Psychology and Norwegian Research Centre of Children and Youth Sports, Norwegian School of Sport Sciences, Oslo, Norway; ^3^Center of Research on Welfare, Health and Sport (CVHI), Halmstad University, Halmstad, Sweden

**Keywords:** motivation regulations, self-control, exhaustion, youth athletes, Bayesian structural equation modeling, longitudinal mediation models

## Abstract

The depletion of self-control competencies has been explained by an external shift in motivation, and recent research has emphasized that controlled types of motivation and self-control competencies are positively associated with exhaustion in youth athletes. Using the self-determination theory (SDT) and self-control theories, this study examined associations between athletes' motivation, self-control competencies, and exhaustion experiences throughout a competitive season. A total of 321 winter sport youth athletes (173 males, 98 females, and 50 unknown gender; aged 16 to 20 years, *M* = 17.98, *SD* = 0.89) participated in this 10-week longitudinal study, including three time points. Using Bayesian structural equation modeling, associations between athletes' reported level of motivation regulations, self-control, and exhaustion throughout their competitive season were examined in two mediation models. Constructs were associated in a conceptual and consistent manner. Simple mediation models showed credible indirect and direct effects of motivation on exhaustion via self-control within amotivation, and intrinsic, integrated, identified, and external regulation analyses. These credible effects were not replicated in the focused mediation model, when controlling for self-control and exhaustion autoregressive effects. However, direction of effects in both models was consistent and congruent. Findings consistently supported the interplay between motivation and exhaustion via self-control in youth athletes over an important competition period of the year. Autonomous and controlled motivation interacted with self-control and, respectively, predicted perceived exhaustion negatively and positively. Thus, autonomous self-control motives are important in preventing negative sport participation development over time. However, simple and focused mediation models showed different results, suggesting a necessity for accurate considerations of analytical methods chosen to investigate longitudinal mediation. Specifically, future studies need to carefully consider the time interval between measurement time points when investigating changes in dynamic psychological constructs, and include autoregressive longitudinal effects in order to predict change in levels of the outcome over time.

## Introduction

In the development of elite performance, young athletes are continuously challenged by social, psychological, and physical demands. In this situation, their motivation and self-control play an important role in their confidence (Martinent and Decret, 2015; Toering and Jordet, 2015). Ideally, athletes' self-control capacity is optimal, directing performance through difficult periods without negative consequences (Gould and Whitley, 2009; Collins and MacNamara, 2012). Based on previous research findings suggesting an important interaction between motivation and self-control in sport participation outcomes (e.g., Englert and Bertrams, 2015; Jordalen et al., 2016), the current study examined whether athletes' motivation and self-control together may reduce risks of exhaustion throughout a competitive season.

Young athletes face psychological, social, physiological, and educational demands and stressors, and they may lack proper family support attending elite sport colleges far away from home (Martinent and Decret, 2015). In this achievement context, they may participate in activities for pleasure and inherent satisfaction without emphasizing the external control (Ryan and Deci, 2000). However, this intrinsic motivation for sport participation prevalent in young sport participants may shift toward more controlled forms of motivation as the competitive aspects of activities become salient (e.g., winning over others; Jordalen, 2017). According to self-determination theory (SDT; Deci and Ryan, 1985; Ryan and Deci, 2000, 2017), autonomous and controlled types of motivation are distinctively different. Autonomous motivation reflects individuals' self-directed and intentional behavior, whereas controlled motivation is externally controlled and directed by others. SDT is based on the assumption that there are three types of autonomous motivation regulations (i.e., intrinsic, integrated, and identified), two types of controlled motivation regulations (i.e., introjected and external), as well as a non-regulated type of motivation (i.e., amotivation; Ryan and Deci, 2000). These types of motivation are located on a self-determination continuum, reflecting the degree to which individuals have internalized the reason why they participate in an activity. Based on the degree of internalization, athletes will show qualitatively different types of motivation, as they may for example participate in sports for inherent reasons and pleasure, or for external reasons such as winning (Ryan and Deci, 2000). However, different types of motivation to participate in a sport often simultaneously lead to good performances (Chemolli and Gagné, 2014). Especially in elite sport contexts, high levels of autonomous and controlled motivation may be advantageous (Gillet et al., 2012; Jordalen, 2017), and as their motivational profile develops, young athletes' self-control competencies may help them (Briki, 2016).

Elite athletes' self-control competencies explicitly and implicitly affect performance outcomes (Toering and Jordet, 2015; Englert, 2017). For example, self-control may help athletes focus on performance improvements and goal achievement during strenuous physical exercise, follow their pre-determined exercise planning, and perform under pressure (Englert, 2017). Self-control defines individuals' capacity to override or alter predominant response tendencies and corresponds to their ability to resist temptations, stay focused, and reframe thoughts during training sessions and competitions (Tangney et al., 2004). This volitional ability is the deliberate, conscious, and effortful subset of individuals' more general self-regulation capacity, akin to willpower and delay of gratification (Baumeister et al., 2007). Even though trait measures as opposed to state measures of self-control is relatively stable and rather individually differentiated, the strength model suggests that subsequent acts of both trait and state self-control may impair its capacity just as a muscle gets tired from exertion (i.e., ego-depletion; Baumeister et al., 2007; de Ridder et al., 2012). However, during the last decade researchers disagree whether ego-depletion effects exist (e.g., see Hagger et al., 2010b; Carter et al., 2015; Cunningham and Baumeister, 2016; Friese et al., 2018). For example, ego-depletion research findings are inconsistent when investigating trait self-control as a moderator (Friese et al., 2018), as they emphasize both more (Imhoff et al., 2014) and less (DeWall et al., 2007) susceptibility to ego depletion effects. Mediator studies investigating ego-depletion are less prevalent, though, these studies are more consistent and findings verify a true ego depletion effect larger than zero (Friese et al., 2018). However, competing models that seek to explain apparent regulatory failure and reduced self-control performance, such as the process model of depletion, have been suggested (Inzlicht and Schmeichel, 2012). In this model, self-control is not resource-based. Subsequent to regulatory failures, a refractory period may emerge due to dynamically changing priorities, motives, and values (Inzlicht et al., 2014; Inzlicht and Marcora, 2016). According to this model, athletes have motivational difficulties staying on track when they execute subsequent acts of self-control, and consequently look for tasks that are intrinsically satisfying and require less cognitive resources (leading to self-control failure; Inzlicht et al., 2014). This process leads to a shift in motivation away from expending effort (e.g., perform the exact number of planned repetitions in a workout) toward focusing on relaxation (e.g., engage in less effortful tasks). As such, associations between motivation and self-control strength are especially relevant in the strenuous surroundings of young elite level athletes. These athletes feel they need to control their activities, and often experience external stressors simultaneously, such as media exposure, travel, and the need to make tough decisions (Jordalen, 2017). Thus, the elite sport context is volitionally demanding, and athletes are especially vulnerable for self-control depletion and exhaustion, increasing the risk for more severe conditions such as athlete burnout (Baumeister et al., 2007; Gould and Whitley, 2009).

Athlete burnout has been conceptualized as a multidimensional psychological syndrome, consisting of emotional and physical exhaustion, reduced sense of accomplishment, and sport devaluation (Raedeke, 1997). Operationally defined, it encompasses a psychophysiological and dysfunctional response to training and competition (Gustafsson et al., 2011), and has been linked to athletes' self-control (Coakley, 1992). External control experienced by young athletes (e.g., constrains from coaches, uncertain future opportunities), may result in a unidimensional sport identity where athletes solely focus on elite sport development. Thus, combined with autonomous and controlled types of motivation, self-control competencies may increase and decrease athletes exhaustion experiences, respectively (Jordalen et al., 2016). Furthermore, self-control provides volitional resources to athletes' autonomous motivation throughout their career trajectories, especially when experiencing challenging periods and negative sport participation outcomes (Jordalen, 2017). When experiencing burnout, they may manage to increase their competencies to function more autonomously when probed to focus on their self-regulation and self-control (e.g., self-management, coping strategies; Dubuc-Charbonneau and Durand-Bush, 2015). Thus, being externally controlled seems to provoke exhaustive sport participation experiences, and athletes' self-control combined with increased feelings of autonomous motivation may help handle and also avoid the aforesaid outcome (Li et al., 2013). Exhaustion has been viewed as the core dimension of burnout (Gustafsson et al., 2016; Lundkvist et al., 2017), and based on the self-control depletion literature (e.g., Baumeister and Vohs, 2016) the current study investigated exhaustion experiences of athlete burnout related to athletes' motivation and self-control competencies.

In sum, internalizing the reasons for participation in sport and experiencing high levels of autonomous motivation and self-control competencies, athletes evidently develop successfully. Likely driven by a combination of internal and external motives, their ensuing motivation will energize activities and serve cognitive and volitional competencies in different ways (Ryan and Deci, 2000; Englert and Bertrams, 2015; Baumeister, 2016). Throughout a competitive season, athletes' motivation may change (Ryan and Deci, 2000; Lemyre et al., 2006), though their trait self-control related to practice sessions and competitions is individually determined, relatively stable, and when autonomously motivated may counteract the deleterious effects of self-control failure and depletion (Hagger et al., 2010a; Anusic and Schimmack, 2016). However, the associations between and functionality of athletes' various types of motivation and self-control competencies have not been investigated in-depth and over an important competitive period. Depending on the degree of autonomous motivation, cross-sectional research emphasizes that athletes' use of self-control may lead to exhaustion (Jordalen et al., 2016; see also Tuk et al., 2015). The overwhelming amount of cross-sectional research investigating mediation effects between psychological constructs does introduce constrained knowledge about the true causation investigated between those constructs, and to examine a corresponding theoretical process model, research must rely on longitudinal data (Maxwell and Cole, 2007; Jose, 2016; Stenling et al., 2017). In addition, studies explicitly examining mediator effects of self-control on depletion hardly exist, even though a statistical mediation reveals indirect evidence for true depletion effects larger than zero (Friese et al., 2018). Thus, the association between motivation and exhaustion over time, mediated by athletes' self-control competencies, was examined throughout an important competitive period in the current study. It was hypothesized that we would find (a) negative associations between autonomous motivation and exhaustion, and (b) positive associations between controlled motivation and exhaustion, and that (c) self-control would mediate these relationships over time.

## Materials and Methods

### Participants

A total of 321 winter sport athletes (173 males, 98 females; 16–20 years of age, *M* = 17.98, *SD* = 0.89) attending elite sport colleges in Norway consented to participate^1^. Participants provided written informed consent in accordance with the Declaration of Helsinki. Athletes competed in cross-country skiing (*n* = 122), biathlon (*n* = 64), ski jumping (*n* = 15), alpine skiing (*n* = 63), and Nordic combined (*n* = 7). They had 1–16 years of competitive experience (CE; *M* = 7.86 years, *SD* = 2.93), and competed at international (*n* = 54), national (*n* = 193), or regional levels (*n* = 24). Descriptive information was collected at T1. Athletes who only participated at T2 and/or T3 (*n* = 50) did not report this information (T1 *n* = 271; T2 *n* = 201; and T3 *n* = 197). Thus, some participants participated at T1 and T2 (*n* = 184), and one-half participated at all-time points (*n* = 136). Analyzing missing data, we performed Bayesian *t*-tests to compare the baseline scores for athletes who participated at all-time points, and athletes who did not participate at all-time points. Results from these *t*-tests showed stronger support for the null hypothesis than for the alternative hypothesis, indicating no statistical differences between the two groups (i.e., Bayes factors for the alternative hypothesis ranged from 0.14 to 1.31). The data was, therefore, treated as missing at random (MAR; Enders, 2010). However, Bayesian estimation using MCMC algorithms with the Gibbs sampler in the current statistical analyses randomly drew parameter values in the posterior distribution to reflect participants' responses (Depaoli and van de Schoot, 2015).

### Measures

#### Motivation

A Norwegian version (Jordalen et al., 2016) of the Sport Motivation Scale II (SMS-II; Pelletier et al., 2013) measured athletes' motivation regulations. The stem prior to presenting each item was “Report the extent to which the listed reasons for practicing your sport corresponds with your own personal reasons during the last month,” and response options ranged from 1 (*does not correspond at all*) to 7 (c*orresponds completely*). Composite reliability (Rho [ρ]; Raykov, 2009) and factor scores' validity coefficients (Brown, 2006) provided reliability information for each subscale (see Table 1). Each assessed regulation included three items, and the regulations were intrinsic (e.g., “because it is very interesting to learn how I can improve”), integrated (e.g., “because participating in sport is an integral part of my life”), identified (e.g., “because I have chosen this sport as a way to develop myself”), introjected (e.g., “because I feel better about myself when I do”), external (e.g., “because people around me reward me when I do”), and amotivated (e.g., “it is not clear to me anymore; I don't really think my place is in sport”).

**Table 1:** Descriptive statistics and correlations of study variables at time point one, two, and three.

	***M***	***SD***	**Rho [ρ]**	**95% CI**	**1**.	**2**.	**3**.	**4**.	**5**.	**6**.	**7**.	**8**.	**9**.	**10**.	**11**.	**12**.	**13**.	**14**.	**15**.	**16**.	**17**.	**18**.	**19**.	**20**.	**21**.	**22**.	**23**.	**24**.
1. InT1	6.17	0.69	0.71	[0.64, 0.76]	0.95	0.62^*^	0.53^*^	0.51^*^	0.37^*^	0.35^*^	0.51^*^	0.35^*^	0.38^*^	0.19^*^	0.10	0.09	0.03	−0.11	−0.01	−0.24^*^	−0.36^*^	−0.24^*^	0.22^*^	0.32^*^	0.19	−0.22	−0.21	−0.17
2. InT2	6.10	0.82	0.80	[0.75, 0.84]		0.99	0.71^*^	0.40^*^	0.66^*^	0.49^*^	0.36^*^	0.64^*^	0.53^*^	0.03	0.17	0.19	−0.01	0.01	0.05	−0.44^*^	−0.47^*^	−0.59^*^	0.33^*^	0.28^*^	0.37^*^	−0.31^*^	−0.28^*^	−0.35^*^
3. InT3	5.95	0.89	0.82	[0.77, 0.85]			0.96	0.32^*^	0.46^*^	0.60^*^	0.30^*^	0.46^*^	0.65^*^	0.11	0.10	0.30^*^	−0.03	−0.10	0.01	−0.29^*^	−0.49^*^	−0.48^*^	0.20	0.30^*^	0.31^*^	−0.23^*^	−0.21	−0.24^*^
4. IeT1	5.80	0.81	0.65	[0.54, 0.72]				1.01	0.65^*^	0.53	0.49^*^	0.44^*^	0.43^*^	0.45^*^	0.35^*^	0.31^*^	0.17	0.06	0.11	−0.17	−0.24^*^	−0.12	0.22^*^	0.20	0.10	−0.12	−0.16	−0.18
5. IeT2	5.79	0.90	0.73	[0.66, 0.78]					1.11	0.68^*^	0.33^*^	0.62^*^	0.52^*^	0.27^*^	0.44^*^	0.39^*^	0.07	0.12	0.16	−0.32^*^	−0.36^*^	−0.38^*^	0.36^*^	0.26^*^	0.28^*^	−0.28^*^	−0.29^*^	−0.41^*^
6. IeT3	5.66	0.93	0.71	[0.64, 0.78]						1.00	0.22	0.36^*^	0.58^*^	0.14	0.20	0.46^*^	−0.05	−0.10	0.03	−0.27^*^	−0.45^*^	−0.45	0.23	0.23	0.32^*^	−0.23	−0.21	−0.36^*^
7. IdT1	5.54	0.96	0.74	[0.68, 0.78]							0.93	0.59^*^	0.43^*^	0.36^*^	0.22	0.26^*^	0.21^*^	0.10	0.20	−0.06	−0.14	−0.14	0.10	0.21	0.11	−0.13	−0.17	−0.06
8. IdT2	5.57	1.04	0.82	[0.77, 0.86]								0.96	0.64^*^	0.22	0.34^*^	0.26^*^	0.16	0.19	0.17	−0.17	−0.26^*^	−0.36^*^	0.20	0.27^*^	0.25	−0.24^*^	−0.28^*^	−0.29^*^
9. IdT3	5.52	0.98	0.83	[0.79, 0.86]									0.95	0.14	0.21	0.33^*^	0.04	−0.01	0.09	−0.21	−0.41^*^	−0.29	0.14	0.27^*^	0.14	−0.29^*^	−0.26^*^	−0.26^*^
10. IrT1	4.45	1.25	0.70	[0.63, 0.77]										0.99	0.67^*^	0.57^*^	0.44^*^	0.30^*^	0.32^*^	0.19^*^	0.12	0.22	−0.13	−0.10	−0.22	0.13	0.09	0.10
11. IrT2	4.37	1.30	0.69	[0.62, 0.76]											1.06	0.67^*^	0.36^*^	0.48^*^	0.39^*^	0.04	0.01	0.11	−0.09	−0.09	−0.16	−0.03	0.06	−0.04
12. IrT3	4.46	1.22	0.68	[0.58, 0.74]												1.01	0.31^*^	0.27^*^	0.40^*^	0.05	−0.02	−0.02	0.00	−0.04	−0.05	0.04	0.11	0.04
13. ET1	2.76	1.17	0.0.66	[0.59, 0.72]													0.94	0.67^*^	0.61^*^	0.29^*^	0.16	0.25^*^	−0.26^*^	−0.22	−0.20	0.30^*^	0.25^*^	0.20
14. ET2	2.73	1.23	0.72	[0.64, 0.77]														0.96	0.61^*^	0.22	0.26^*^	0.26^*^	−0.30^*^	−0.34^*^	−0.30	0.21	0.24^*^	0.18
15. ET3	2.86	1.17	0.71	[0.65, 0.77]															0.95	0.14	0.21	0.27^*^	−0.12	−0.21	−0.24^*^	0.17	0.16	0.32^*^
16. AT1	2.36	1.44	0.84	[0.80, 0.87]																1.06	0.75^*^	0.64^*^	−0.29^*^	−0.31^*^	−0.37^*^	0.44^*^	0.32^*^	0.34^*^
17. AT2	2.43	1.55	0.87	[0.83, 0.89]																	1.01	0.81^*^	−0.36^*^	−0.46^*^	−0.55^*^	0.45^*^	0.52^*^	0.52^*^
18. AT3	2.61	1.59	0.87	[0.83, 0.90]																		0.94	−0.31^*^	−0.36^*^	−0.58^*^	0.43^*^	0.50^*^	0.59^*^
19. ScT1	3.56	0.52	0.78	[0.75, 0.82]																			0.96	0.74^*^	0.63^*^	−0.49^*^	−0.47^*^	−0.31^*^
20. ScT2	3.57	0.54	0.80	[0.72, 0.84]																				0.95	0.71^*^	−0.50^*^	−0.51^*^	−0.43^*^
21. ScT3	3.44	0.52	0.82	[0.78, 0.86]																					0.96	−0.40^*^	−0.41^*^	−0.49^*^
22. ExT1	2.13	0.76	0.87	[0.85, 0.90]																						0.97	0.61^*^	0.60^*^
23. ExT2	2.05	0.72	0.86	[0.83, 0.89]																							0.98	0.65^*^
24. ExT3	2.20	0.78	0.89	[0.86, 0.91]																								0.96

*In, Intrinsic regulation; Ie, Integrated regulation; Id, Identified regulation; Ir, Introjected regulation; E, External regulation; A, Amotivation regulation; Ex, Exhaustion; T1, T2, T3, time point one, two, three; Rho [ρ], Composite reliability; CI, Credibility interval*.

*Factor scores' validity coefficients at the diagonal [recommended score >0.80; (Brown, 2006)]*.

* *BF >10*.

#### Trait Self-Control (SC)

A Norwegian version (Toering and Jordet, 2015) of the Brief Self-Control Scale (BSCS; Tangney et al., 2004) assessed athletes' dispositional SC abilities (13 items, e.g., “I am good at resisting temptations”). The stem prior to presenting each item was “Please indicate how much each of the following statements reflect your typical thoughts and actions during the last month,” and response options ranged from 1 (*not at all*) to 5 (*very much*). Items 2–5, 7, 9, 10, 12, and 13 were reverse scored (Tangney et al., 2004). Items 6 and 8 were deleted due to low factor loadings (< 0.20; Kline, 2011).

#### Exhaustion

A Norwegian version (Lemyre et al., 2006) of the Athlete Burnout Questionnaire (ABQ; Raedeke and Smith, 2001) assessed athletes' experiences of physical and emotional exhaustion (five items, e.g., “I feel'wiped out' from [sport]”). The stem prior to presenting each item was “How often have you experienced the following statements regarding your sport participation during the last month,” and response options ranged from 1 (*almost never*) to 5 (*almost always*).

### Procedures

Subsequent to approval by the Norwegian Center for Research Data (NSD), national ethical standard procedures were followed for the protection of research participants. Following approval from sport directors and coaches at elite sport colleges (recognized by the Norwegian Ski Federation), athletes were invited to participate. The first author held written and verbal presentations of the study, and visited colleges every fifth week for data collection, three times in total. Athletes agreeing to participate provided written informed consent. Answering questionnaires, they indicated the extent to which questions reflected their sport related thoughts and actions during the previous month. The data collection period was arranged in the middle of athletes' competitive season and included important events—such as national and international competitions and college exams—that challenge young athletes socially, psychologically, and physiologically. SurveyXact version 8.0 (QuickQuest) was used to collect data.

### Statistical Analyses

Descriptive statistics, variables composition, model fit, and reliability were examined in M*plus* 7.4 (Muthén and Muthén, 1998-2016; Raykov, 2009), and correlation analysis was performed in JASP 0.8.0.0 (see Table 1). Further, six structural equation mediation models were analyzed using the Bayesian estimator in M*plus* 7.4 (Muthén, 2010), thus each model corresponded to one motivation regulation. Investigating causal mechanisms between variables over time, research often relies on cross-sectional data that does not include the temporality criteria of causation (Cole and Maxwell, 2003; Jose, 2016; Stenling et al., 2017). However, the current study's longitudinal design incorporated this foundational causal mechanism of mediation (i.e., the time-lagged influence of variables; Jose, 2016; Stenling et al., 2017), and investigated previous cross-sectional findings (Jordalen et al., 2016).

**Figure 1:**
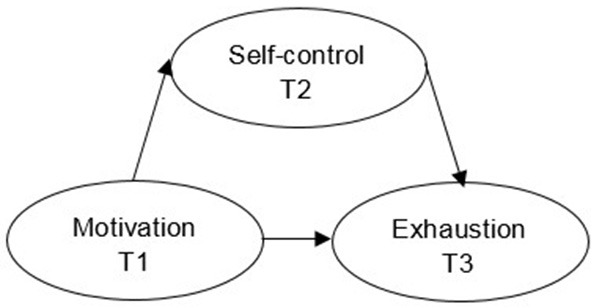
The structural simple longitudinal mediation model. T1, T2, and T3 = time point one, two, and three.

**Figure 2:**
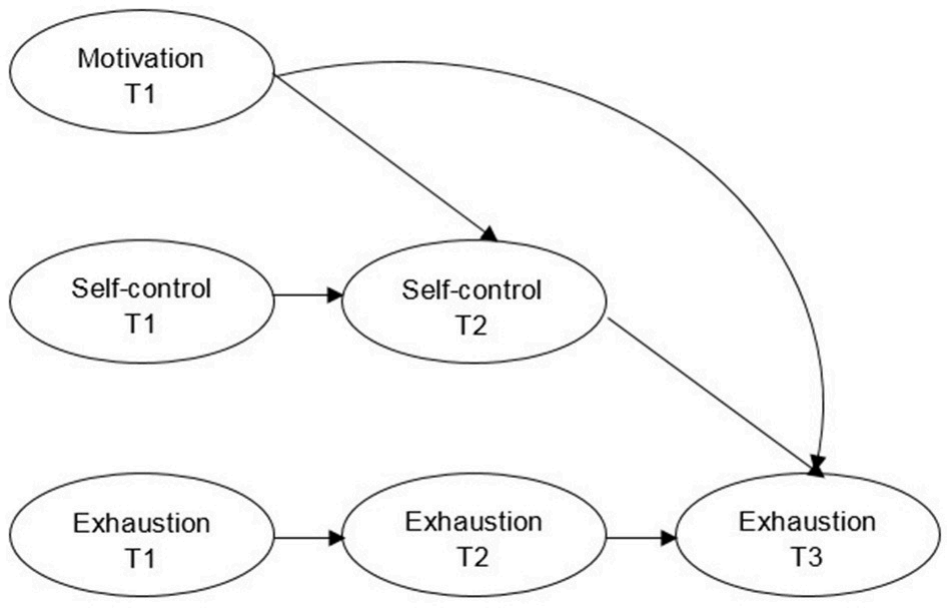
The structural focused longitudinal mediation model. T1, T2, and T3 = time point one, two, and three.

First, a simple mediation model was used to investigate the hypothesized ordering of variables (Figure 1; Selig and Preacher, 2009). The model was used to look at causal associations at three time points, specifically the effect of T1 motivation on T3 exhaustion via T2 self-control. In these analyses, those aspects of the model that influenced the outcome variable were investigated (Jose, 2016). However, this model did not control for prior assessments of the mediator and the outcome variable (i.e., autoregressive effects), and may imply indirect and direct effects erroneously (Cole and Maxwell, 2003). Further, using this mediation model to investigate variables may lead to a simplified worldview that does not consider dynamic social and psychological characteristics (Gelman, 2015). These shortcomings in mediation analyses have been described the last 20 years, but the majority of mediation analyses still rely on cross-sectional analyses (Maxwell et al., 2011; Tate, 2015; Trafimow, 2015; e.g., see Zhang et al., 2016). As such, a focused mediation model was performed, controlling for autoregressive effects testing the specific ordering of variables (Figure 2). Specifically, this model investigated whether T1 motivation increased or decreased T2 self-control, and whether T2 self-control increased or decreased T3 exhaustion. In the simple and focused mediation models, total effects are reported as the unmediated associations between motivation and exhaustion, direct effects as the mediated associations between motivation and exhaustion, and indirect effects as the estimated effect of self-control in the motivation to exhaustion association (Jose, 2016).

Based on the Bayes' theorem, Bayesian Structural Equation Modeling (BSEM) uses a combination of information (priors) from previous studies and the current data to generate the posterior distribution (Muthén and Asparouhov, 2012). Compared to the more traditional ML estimation (i.e., frequentist statistics), this approach aids in model identification, improves convergence issues, and is advantageous when researchers deal with small sample sizes (Depaoli and van de Schoot, 2015). Parameter specifications of exact zeroes are replaced with approximate zeros by weakly informative priors that influence the posterior distribution to a lesser extent (Muthén and Asparouhov, 2012; Depaoli and van de Schoot, 2015). Current analysis' priors allowed for small variances in the simple mediation analyses and small cross-loadings and variances in the focused mediation analyses, within and between each latent variable at different time points.

We implemented Bayesian models using two Markov Chain Monte Carlo (MCMC) simulation procedures with the Gibbs sampler (Depaoli and van de Schoot, 2015). A potential scale reduction factor around 1 indicated convergence (Muthén and Asparouhov, 2012), and convergence cut-off values specified to 0.01 reduced bias caused by precision (van de Schoot et al., 2013, 2014). All models were run with 200,000 iterations (100,000 burn-in by default), and every 10th iteration was used to reduce autocorrelation between MCMC draws (De Bondt and Van Petegem, 2015). Trace plots were inspected visually to determine chain convergence (available upon request; Depaoli and van de Schoot, 2015). The posterior predictive *p* (PP*p*) value in combination with the 95% confidence interval (CI^2^) reflected model fit (van de Schoot et al., 2014). A PP*p* close to.50 and a symmetric 95% CI^2^ centering on zero indicate excellent fit (Muthén and Asparouhov, 2012). Additionally, Bayesian Information Criteria (BIC) and deviance information criterion (DIC) were used to compare nested models, and lower values indicated a better fitting model (Asparouhov et al., 2015). In each model, parameters' CI indicate the probability that the current parameters' “true value” is between the two values given the observed data. The null hypothesis was rejected if the 95% CI did not include zero (Zyphur and Oswald, 2015).

In the simple and focused mediation analyses, small-variance informative priors were specified for the residual correlations (IW = 0, 50). Few studies have investigated the longitudinal relationships between current study variables, and there were no reliable research findings on which to base a prior specification. Thus, non-informative priors for the structural parameter estimates were used in both analyses (Yuan and MacKinnon, 2009). However, small-variance priors for cross-loadings between variables measured at the same time point were specified in the focused mediation models (0, 0.005). These focused models included variables at each time point, and measurement invariance was specified (Little, 2013). This method evaluates whether constructs are exact equivalent over time, and respondents attribute the same meaning to the latent factor(s) and equality in the levels of underlying items at different time points (van de Schoot et al., 2012). Building on the current study's BSEM analyses, approximate measurement invariance (AMI) tested constructs approximate equivalence over time (van de Schoot et al., 2013). That is, a two-step approach tested factor loading and intercept parameters AMI simultaneously, freeing non-invariant parameters in the second step (Muthén, and Asparouhov, 2013). This method allows for some wiggle room for factor loadings and intercept variance differences between time points, as the precision of priors may vary. AMI was specified with small-variance informative priors (0, 0.01)^3^.

## Results

### Descriptive Statistics

Descriptive statistics and correlations are presented in Table 1. In the BSEM analyses, the models' complexity was reduced by evaluating motivation regulations individually in six different mediation model analyses (Kline, 2011). Recent research findings argue that investigating the specific motivation regulations will provide detailed information beyond investigating one global factor of motivation (e.g., using a relative autonomy index; Chemolli and Gagné, 2014; Howard et al., 2016; Litalien et al., 2017). Meeting indicator requirements for one-factor models (Brown, 2006), three, five, and eleven indicators were specified defining motivation regulations, exhaustion, and self-control latent constructs, respectively. These model specifications showed good fit to the data (see Tables 2 and 3), and analyses confirmed acceptable reliability and validity coefficients (see Table 1). In general, athletes reported high levels of self-control and intrinsic, integrated, and identified regulations; moderate levels of introjected regulation and exhaustion; and low levels of external regulation and amotivation. Throughout the season, they reported decreased levels of autonomous motivation and self-control and increased levels of controlled motivation and exhaustion.

**Table 2:** Standardized estimates for structural paths, simple mediation models.

	**Intrinsic**		**Integrated**		**Identified**		**Introjected**		**Extrinsic**		**Amotivation**	
	**Est**.	**95%CI**	**Est**.	**95%CI**	**Est**.	**95% CI**	**Est**.	**95%CI**	**Est**.	**95%CI**	**Est**.	**95% CI**
**STRUCTURAL PATHS**												
MotT1 – ScT2 (a)	0.29	[0.14,0.42]	0.20	[0.02, 0.35]	0.24	[0.06, 0.39]	−0.14	[−0.29, 0.03]	−0.22	[−0.36, −0.06]	−0.27	[−0.41, -0.11]
ScT2 – ExT3 (b)	−0.44	[−0.58, −0.28]	−0.43	[−0.57, −0.28]	−0.48	[−0.61, −0.31]	−0.43	[−0.56, −0.26]	−0.42	[−0.55, −0.26]	−0.38	[−0.53, −0.21]
MotT1 – ExT3 (c')	−0.04	[−0.20, 0.11]	−0.09	[−0.23, 0.06]	0.06	[−0.09, 0.22]	0.09	[−0.06, 0.23]	0.14	[−0.02, 0.28]	0.28	[0.14, 0.42]
Total effect (c)	−0.17	[−0.32, −0.02]	−0.17	[−0.31, −0.02]	−0.05	[−0.21, 0.11]	0.14	[−0.01, 0.29]	0.23	[0.07, 0.37]	0.39	[0.24, 0.51]
Indirect effect (a*b)	−0.12	[−0.21, −0.05]	−0.08	–[0.17, −0.01]	−0.11	[−0.21, −0.03]	0.06	[−0.01, 0.13]	0.09	[0.02, 0.17]	0.10	[0.04, 0.17]
**VARIANCE EXPLAINED**												
R^2^-ScT2	0.08	[0.02, 0.18]	0.04	[0.01, 0.13]	0.06	[0.01, 0.16]	0.02	[0.00, 0.09]	0.05	[0.01, 0.13]	0.07	[0.01, 0.17]
R^2^-ExT3	0.21	[0.10, 0.34]	0.22	[0.10, 0.35]	0.22	[0.10, 0.36]	0.21	[0.09, 0.34]	0.23	[0.10, 0.36]	0.29	[0.16, 0.42]
**MODEL FIT INDICES**												
PP*p*	0.27		0.23		0.22		0.22		0.25		0.25	
95% CI^2^	[−39.82, 78.25]		[−35.66, 87.46]		[−35.20, 82.59]		[−35.47, 82.74]		[−36.82, 87.50]		[−37.15, 84.13]	

*MotT1, Motivation T1 (for specific type of motivation see table); ScT2, Self-control T2; ExT3, Exhaustion T3; Total effect, Total effect predictor on dependent variable; Indirect effect, Indirect effect of Motivation T1 on Exhaustion T3 via Self-control T2; T1, T2, and T3, time point one, two, and three; Est., Estimate; CI, Credibility interval; CI^2^, Confidence interval; PPp, Posterior predictive p-value*.

**Table 3:** Standardized estimates for structural paths, focused mediation models.

	**Intrinsic**		**Integrated**		**Identified**		**Introjected**		**Extrinsic**		**Amotivation**	
	**Est**.	**95% CI**	**Est**.	**95% CI**	**Est**.	**95 % CI**	**Est**.	**95% CI**	**Est**.	**95% CI**	**Est**.	**95 % CI**
**AUTOREG. EFFECTS**												
ScT1-ScT2	0.61	[0.49, 0.70]	0.54	[0.54, 0.72]	0.63	[0.53, 0.71]	0.63	[0.53, 0.71]	0.64	[0.53, 0.73]	0.63	[0.53, 0.72]
ExT1-ExT2	0.57	[0.46, 0.66]	0.57	[0.46, 0.65]	0.56	[0.45, 0.65]	0.56	[0.46, 0.65]	0.56	[0.45, 0.65]	0.57	[0.46, 0.65]
ExT2-ExT3	0.57	[0.44, 0.67]	0.57	[0.44, 0.67]	0.58	[0.45, 0.68]	0.56	[0.44, 0.67]	0.56	[0.43, 0.67]	0.54	[0.40, 0.65]
**STRUCTURAL PATHS**												
MotT1 – SoT2 (a)	0.11	[−0.03, 0.24]	0.03	[−0.11, 0.17]	0.11	[−0.04, 0.26]	−0.06	[−0.20, 0.08]	0.01	[−0.14, 0.14]	−0.07	[−0.21, 0.08]
ScT2 – ExT3 (b)	−0.16	[−0.32, 0.01]	−0.17	[−0.32, −0.01]	−0.21	[−0.36, −0.05]	−0.18	[−0.33, −0.02]	−0.18	[−0.33, −0.02]	−0.15	[−0.30, 0.01]
MotT1 – ExT3 (c')	−0.08	[−0.23, 0.06]	−0.11	[−0.25, 0.01]	0.05	[−0.09, 0.19]	0.10	[−0.03, 0.23]	0.09	[−0.05, 0.23]	0.15	[0.01, 0.29]
Total effect (c)	−0.11	[−0.24, 0.04]	−0.12	[−0.25, 0.01]	0.03	[−0.11, 0.16]	0.12	[−0.02, 0.24]	0.09	[−0.05, 0.23]	0.16	[0.01, 0.30]
Indirect effect (a*b)	−0.02	[−0.05, 0.01]	−0.003	[−0.04, 0.02]	−0.02	[−0.07, 0.01]	0.01	[−0.02, 0.04]	−0.01	[−0.04, 0.04]	0.01	[−0.02, 0.04]
**CORRELATIONS**												
MotT1—ScT1	0.27	[0.06, 0.46]	0.26	[0.05, 0.45]	0.14	[−0.05, 0.33]	−0.16	[−0.34, 0.03]	−0.27	[−0.43, −0.09]	−0.32	[−0.46, −0.15]
MotT1—ExT1	−0.27	[−0.47, −0.06]	−0.18	[−0.38, 0.03]	−0.16	[−0.35, 0.03]	0.16	[−0.02, 0.33]	0.32	[0.15, 0.47]	0.45	[0.30, 0.58]
ScT1—ExT1	−0.52	[−0.65, −0.37]	−0.54	[−0.65, −0.40]	−0.54	[−0.65, −0.40]	−0.54	[−0.65, −0.40]	−0.52	[−0.64, −0.37]	−0.53	[−0.65, −0.38]
ScT2—ExT2	−0.14	[−0.35, 0.09]	-0.14	[−0.36, 0.09]	−0.12	[−0.34, 0.11]	−0.14	[−0.35, 0.09]	−0.14	[−0.36, 0.09]	−0.15	[−0.37, 0.08]
**VARIANCE EXPLAINED**												
R^2^-ScT2	0.42	[0.30, 0.53]	0.42	[0.31, 0.52]	0.43	[0.32, 0.53]	0.42	[0.30, 0.52]	0.41	[0.29, 0.52]	0.43	[0.32, 0.54]
R^2^-ExT2	0.32	[0.21, 0.43]	0.32	[0.21, 0.42]	0.32	[0.21, 0.42]	0.32	[0.21, 0.43]	0.31	[0.21, 0.42]	0.32	[0.21, 0.42]
R^2^-ExT3	0.44	[0.32, 0.55]	0.45	[0.33, 0.56]	0.44	[0.32, 0.55]	0.44	[0.32, 0.55]	0.44	[0.33, 0.55]	0.44	[0.32, 0.55]
**MODEL FIT INDICES**												
PP*p*	54		52		54		53		49		51	
95% CI^2^	[−120.34, 112.64]		[−124.62, 110.44]		[−119.01, 116.23]		[−121.54, 113.48]		[−120.65, 120.23]		[−124.29, 114.11]	

*MotT1, Motivation T1 (for specific type of motivation see table); ScT1, Self-control T1; ScT2, Self-control T2; ExT1, Exhaustion T1; ExT2, Exhaustion T2; ExT3, Exhaustion T3; Autoreg. effects, autoregressive effects; Total effect, Total effect of predictor on dependent variable; Indirect effect, Indirect effect of Motivation T1 on Exhaustion T3 via Self-control T2; T1, T2, and T3, time point one, two, and three; Est, Estimate; CI, Credibility interval; CI^2^, Confidence interval; PPp, Posterior predictive p-value*.

### Simple Mediation Analyses

The six simple mediation models showed good data-model fit (e.g., PP*p* range: 0.22–0.25; see Table 2). In these analyses, indirect effects between T1 motivation and T3 exhaustion via T2 self-control were negative and credible in the intrinsic (β = −0.12, 95% CI [−0.21, −0.05]) integrated (β = −0.08, 95% CI [−0.17, −0.01]), and identified (β = −0.11, 95% CI [−0.21, −0.03]) regulation analyses. In addition, indirect effects were positive and credible in the external regulation (β = 0.09, 95% CI [0.02, 0.17]) and amotivation (β = 0.10, 95% CI [0.04, 0.17]) analyses. Further, total effects in the intrinsic (β = −0.17, 95% CI [−0.32, −0.02]) and integrated (β = −0.17, 95% CI [−0.31, −0.02]) regulation analyses were negative and credible, though positive and credible in the external regulation (β = 0.23, 95% CI [0.07, 0.37]) and amotivation (β = 0.39, 95% CI [0.24, 0.51]) analyses. No credible direct effects except T1 amotivation to T3 exhaustion (β = 0.28, 95% CI [0.14, 0.42]) were found. In addition, a small and medium amount of variance explained T2 self-control and T3 exhaustion (*R*^2^s = 0.02–0.08; and *R*^2^s = 0.21–29; respectively).

### Focused Mediation Analyses

The six focused mediation models indicated good data-model fit (e.g., PP*p* range: 0.49 to 0.54; see Table 3). These models reflected strong positive, credible autoregressive effects for both self-control (βs = 0.61–0.63) and exhaustion (βs = 0.54–0.57). Further, indirect effects between T1 motivation and T3 exhaustion via T2 self-control, were not found, but the total effect between T1 amotivation and T3 exhaustion was positive and credible (β = 0.16, 95% CI [0.01, 0.30]). Additionally, variables were credibly related within and between time points (Table 3); and substantial amounts of variance explained T2 self-control (*R*^2^s = 0.41–0.43), and T2 and T3 exhaustion (*R*^2^s = 0.32, and *R*^2^s = 0.41–0.43, respectively).

## Discussion

This study investigated the causal chain between six types of motivation regulation (Ryan and Deci, 2000), self-control (Baumeister et al., 2007), and physical and emotional exhaustion in youth athletes throughout a competitive 10-week period. More specifically, the hypothesized mediation effect of self-control on the relationships between six types of motivation and exhaustion was tested in simple and focused mediation models (Jose, 2016). The dynamic nature of these psychological phenomena was examined longitudinally applying Bayesian methods (Muthén and Asparouhov, 2012), accounting for parameter estimates' uncertainty and variation, and addressing their non-constant measures of reality (Gelman, 2015). This approach acknowledges that athletes' responses might be influenced by a variety of factors not measured, and further they may change over time and according to the situation. Hence, parameter estimates are not considered true or false, but credible or non-credible (i.e., there is a 95% probability that the parameter estimate of interest falls within the interval limits given the observed data; van de Schoot et al., 2014).

Athletes' perceptions of self-control and exhaustion were conceptually and consistently associated with autonomous and controlled motivation (e.g., Lemyre et al., 2007; Muraven, 2008), as were the negative associations between self-control and exhaustion (e.g., Baumeister et al., 2007; Seibert et al., 2016). That is, autonomous motivation was positively and negatively associated with self-control and exhaustion, respectively, and controlled motivation was, respectively, negatively and positively associated with self-control and exhaustion. Given these results, it is important that coaches and significant others provide autonomy-supportive climates, accompanied by appropriate structure and involvement, fostering intrinsic and autonomous motivation in the sport context (e.g., Schinke et al., 2017). These results further show that trait self-control is positively and negatively associated with exhaustion experiences dependent on various types of motivation, rather than a sole lack of energy resources, confirming the ego depletion effect (Baumeister and Vohs, 2007; Friese et al., 2018). Autonomous motivation energizes athletes' self-control competencies, for example due to the enhanced subjective value of athletes' goal attainment, thus confirm its' beneficial characteristics postulated in the SDT (Deci and Ryan, 2000; Ryan and Deci, 2000; Berkman et al., 2017). Autonomously motivated, as opposed to controlled motivated athletes, are likely to act in accordance with their values and needs, and unconflicted control their actions in a more flexible manner (Ryan and Deci, 2000). Additionally, the variability in these concepts throughout the 10-week period reflects the importance of longitudinally monitoring youth athletes to influence and facilitate their development (Lemyre et al., 2006; Tuk et al., 2015). However, these preliminary analyses did not examine causality, rather simple, and focused mediation models investigated the causal processes involved (Jose, 2016; Stenling et al., 2017).

In the simple longitudinal mediation model, the mediating effect of self-control on motivation and exhaustion associations was evident in all motivation regulations except introjected regulation. These findings are in line with previous cross-sectional study findings (e.g., Jordalen et al., 2016), and confirm associations between autonomous motivation and self-control (Hagger et al., 2010a). Even though introjected regulation is internal and involves self-control, it is based on feelings of an external perceived locus of causality (Ryan and Deci, 2000). For example, in the competitive elite sport context, athletes may be driven by an internal eagerness to avoid shame and guilt, and simultaneously base their sense of self-worth and self-confidence on externally controlled sport outcomes such as winning over others and attaining status (Holmberg and Sheridan, 2013). Thus, this combination of internal and external motives experienced in introjected regulation merged with athletes' self-control and reflected a positive, though weak and multifaceted, association with exhaustion. When internalizing this introjected type of motivation to be more congruent with an athlete's values, interests, and involvement, he or she will typically experience an internal perceived locus of causality and increased autonomous, and eventually intrinsic, motivation (Ryan and Deci, 2000). Research in competitive sport suggests a complex pattern of motivation in elite athletes, as high levels of autonomous and controlled motivation may simultaneously and positively direct athletes' behaviors (Langan et al., 2016). However, combining internal and external types of motivation may lead to extreme performance levels but may also lead to athletes developing a more fragile motivational profile, putting them at risk for exhaustion, and burnout (Gillet et al., 2012; Chemolli and Gagné, 2014; Martinent and Decret, 2015). Current study findings confirmed a complex pattern of motivation in elite sport participants, showing that each regulation is linked differently to self-control and exhaustion and should thus be investigated separately (Chemolli and Gagné, 2014). Although some associations were barely non-credible, the parameter estimates suggest an important contribution and should not be objectively considered credible or non-credible (Gelman, 2015). Additionally, these analyses did not account for the psychological constructs' dynamic nature, and focused mediation models were performed to examine the change processes involved throughout athletes' competitive season (Gelman, 2015; Jose, 2016).

In the focused mediation models, athletes' initial self-control and exhaustion best predicted subsequent levels of self-control and exhaustion, respectively (Adachi and Willoughby, 2015). Thus, the autoregressive effects confirmed stability of individual differences in the psychological constructs over time (Preacher, 2015; Stenling et al., 2017). Controlling for these stability effects, the focused mediation models showed inter-individual differences in change on the outcome over time, and reflected whether motivation truly predicted change in levels of exhaustion over the 10-week competition period of data collection (Adachi and Willoughby, 2015). Additionally, the strength of autoregressive effects revealed that trait measures of self-control were more stable compared to state measures of exhaustion. These results reflect the relative stability of trait measures across situations and over time (Anusic and Schimmack, 2016), and a requirement to assess the role of time in experiences of exhaustion and the development of burnout (de Ridder et al., 2012; Lundkvist et al., 2017). As athlete burnout is considered enduring, these results confirm that significant time is needed to note changes (e.g., Martinent et al., 2014).

Due to the inclusion of autoregressive paths, effect sizes in the focused mediation models were dramatically smaller compared to effect sizes in the simple mediation models (Adachi and Willoughby, 2015). None of the direct or the indirect effects were credible in the focused mediation model, except the direct effect between amotivation (T1) and exhaustion (T3). Amotivated athletes likely lack a sense of control and efficacy, and presumably experience negative consequences such as exhaustion (Deci and Ryan, 2000). However, the direction of estimates in both models reflected trends in the same direction, and were in line with the hypothesized association between motivation, self-control, and exhaustion. This suggests that athletes' autonomous motivation positively serves their self-control competencies, as they are less prone to experience depletion and exhaustion (Muraven, 2008; Hagger et al., 2010a). Contrary, athletes' controlled types of motivation positively associated with experienced exhaustion via self-control, strengthening a motivational explanation of ego depletion. That is, within the dynamics of psychological constructs and a complex world, not only previous acts of self-control lead to depletion and impaired self-control performance (Fiedler, 2017; Friese et al., 2018). Current study findings thus emphasize that the motivational accounts of ego depletion work in opposite causal directions. Controlled types of motivation seem to evoke processes leading to depletion, whereas self-control performance seems to be triggered by autonomous types of motivation. However, the debate whether ego-depletion exists or not is beyond the scope of this paper (for elaborations, e.g., see Baumeister and Vohs, 2016; Hagger and Chatzisarantis, 2016; Friese et al., 2018). Further, it is important to consider that the 95% CI corresponds to a 95% probability that the parameter estimate of these effects lies within the limits of the interval (van de Schoot et al., 2014). As confirmed in other longitudinal studies (e.g., Curran et al., 2016), causal paths in the focused mediation models were small and non-credible, even though they predicted meaningful proportions of variance close to the autoregressive effects. Therefore, the predictive value of motivation and self-control explaining athletes' exhaustion over a 10-week period was confirmed.

### Limitations

The current study sheds light on associations between motivation and exhaustion via self-control in two mediation models. However, possible limitations concerning questionnaires, design, and type of analyses should be addressed. For example, the content validity of the sport motivation scale has been criticized (Langan et al., 2016). The translated second version of this questionnaire may cause linguistic or cultural misinterpretations (Benítez et al., 2016), and the wording of items may not necessarily apply to elite sport contexts (e.g., asking athletes if they were engaged because they enjoyed learning more about their sport). There is also an ongoing debate concerning the factor structure and the proposed unidimensionality of the BSCS (Tangney et al., 2004), claiming that this is a two-factor scale with various compositions (e.g., Maloney et al., 2012; Toering and Jordet, 2015). In the current analyses, the scale's original unidimensional composition was used. Though, two items were deleted due to low factor loadings (Kline, 2011), and we do question the psychometric properties of the scale. Self-control reverse scored items as well as social desirability may further cause method bias (Podsakoff et al., 2003). Social desirability has often been ignored in studies using self-report measures, even though it causes major threats to the validity of data, secondly influencing associations between variables (Grossbard et al., 2007). It is possible that young athletes respond to questionnaires influenced by social desirability, as they want to present a positive image and appear consistent with their perceived values of for example youth sport programs. The fact that athlete respondents may answer questionnaires to gain social approval or deny negative attributes, for example by their coach or the researcher, may thus contribute to biased results. Further, the investigated effects in the mediation analyses will vary as a function of time and time lag between assessments (Cole and Maxwell, 2003; Preacher, 2015; Anusic and Schimmack, 2016), and the 10-week time frame with three time point measurements may not accurately reflect longitudinal associations in these constructs. Finally, current study analyses examined a specific ordering of variables in simple and focused mediation effects, and did not explore variables' temporal precedence and all possible mediations (i.e., complete mediation; Maxwell et al., 2011; Jose, 2016). Taking these limitations into account findings could have been more persuasive, emphasizing important information in theoretical, and applied research.

### Future Directions

This study aimed to investigate how motivation and self-control together caused athletes' exhaustion, and future research should consider alternative measures that highlight different aspects of these concepts. For example, do athletes' self-control competencies and self-efficacy concerning goal achievement in sport associate with self-control resource depletion, secondly affecting performance, as suggested by Hagger et al. (2010b)? In addition, due to the positive effects of autonomous motivation and self-control competencies, future research should explore how to improve these beneficial psychological characteristics through interventions. Is it possible that self-control resembles a muscle, which will be strengthened by repeated exercise (Baumeister et al., 2007)? Does athletes' self-control increase by providing an autonomy-supportive climate that enhances autonomous motivation (e.g., Berkman et al., 2017)? Is it possible to increase athletes' self-control strength through interventions where athletes and researchers discuss and work through important self-control processes, such as behavioral and emotional responses, self-management, enhanced focus, as well as thought and impulse control (e.g., see Dubuc-Charbonneau and Durand-Bush, 2015)? Further, self-report measures of trait self-control are distinctively different from specific self-control processes (Allom et al., 2016), and high trait self-control ironically shows greater reduction of self-control across dual-task experiments (Imhoff et al., 2014). For example, the brief self-control scale is based on whether participants think they have self-control, and future research should attempt to monitor athletes' actual self-control throughout their everyday endeavors. This would additionally eliminate the risk of social desirability method bias, which is necessary to enhance the validity of the data and subsequent interpretations (Podsakoff et al., 2003; Grossbard et al., 2007). As such, sport psychology research should apply longitudinal designs and methods to explore these causal processes in athletes' everyday life (Preacher, 2015; Stenling et al., 2017). This study especially emphasized autoregressive effects, and longitudinal research should perhaps include three or more waves and consider various measurement intervals to reliably capture long-term reciprocal patterns (Marsh and O'Mara, 2008; Stenling et al., 2017). Additionally, there is a need to develop guidelines for the interpretation of longitudinal mediation models, as current guidelines used to interpret simple mediation models are misleading when controlling for constructs' stability (Adachi and Willoughby, 2015). A full investigation of how these psychological constructs associate and predict change should be examined in a complete mediation model that grasps all possible mediations (Jose, 2016). Finally, a careful investigation of the current study questionnaires in youth sport contexts is warranted to make sure they apply to and grasp athletes' experiences.

## Conclusion

Investigating the causal paths between motivation and exhaustion via self-control competencies, current study findings were inconsistent in two different mediation models. Simple mediation models showed indirect effects within the intrinsic, integrated, identified, and external regulations and amotivation analyses. However, focused mediation models did not replicate these credible findings, but confirmed the longitudinal stability of self-control and exhaustion. However, directions of effects were confirmed in both models. That is, autonomous and controlled motivation on exhaustion via self-control was, respectively, positive and negative, and direct effects from amotivation to exhaustion were positive and credible. These results suggest that high levels of self-control are advantageous when simultaneously driven by autonomous motivation, as athletes are less prone to experience exhaustion over an extensive competition period. Conversely, driven by more controlled motives, athletes high in self-control seem more prone to experience negative sport participation outcomes. Thus, the interaction of motivation and self-control processes should be accurately monitored over time to recognize early symptoms of exhaustion.

## Ethics Statement

This study was carried out in accordance with the recommendations of the Norwegian Center for Research Data (NSD), and national ethical standard procedures were followed for the protection of research participants with written informed consent from all subjects. All subjects gave written informed consent in accordance with the Declaration of Helsinki. The protocol was approved by the Norwegian Center for Research Data (NSD).

## Author Contributions

All authors listed have made a substantial, direct and intellectual contribution to the work, and approved it for publication.

### Conflict of Interest Statement

The authors declare that the research was conducted in the absence of any commercial or financial relationships that could be construed as a potential conflict of interest.

## Footnotes

^1^Athletes in the current longitudinal study arranged during the 2015/16 winter sport season, share similar characteristics with athletes from previous cross-sectional studies (e.g., Jordalen et al., 2016), but the samples do not overlap.

^2^Abbreviations for confidence intervals are marked CI^2^, and abbreviations for credibility intervals are marked CI.

^3^In sensitivity analyses (available upon request), approximate invariance (Normal; 0.001, 0.005, 0.01), cross-loading (Normal; 0.001, 0.005, 0.01), and residual correlation (Inverse Wishart, IW; 100, 500, 33) variances were tested. The various priors did not substantially change mediation model results.
